# From breath to brain: influenza vaccination as a pragmatic strategy for dementia prevention

**DOI:** 10.1007/s40520-026-03323-5

**Published:** 2026-01-16

**Authors:** Lorenzo Blandi, Marco Del Riccio

**Affiliations:** 1 School of Public Health, Vita-Salute San Raffaele University, Via Olgettina 58, Milan, 20132 Italy; 2 Department of Health Sciences, University of Florence, Viale G.B. Morgagni 48, Florence, 50134 Italy

**Keywords:** Influenza vaccination, Dementia, Cognitive decline, Older adults, Vascular dementia, Alzheimer’s disease, Cardiovascular events, Neuroinflammation, Vaccine hesitancy, Public health policy

## Abstract

Aging populations require scalable strategies to delay or prevent dementia. Beyond the prevention of neurological injury associated with seasonal influenza, vaccination may help mitigate vascular and neuroinflammatory injury underlying cognitive impairment. Influenza infection can cause a marked short‑term increase in myocardial infarction risk, and acute infections have also been associated with transient increases in stroke risk. Experimental models show prolonged microglial activation and synaptic loss even from non-neurotropic strains - processes likely modulated by vaccination. Epidemiologic data consistently support this evidence; a 2023 meta-analysis, including observational studies, of ~ 2.09 million adults identified a 31% lower risk of incident dementia; US matched cohorts demonstrated 40% lower risk of Alzheimer’s disease (absolute decrease 3.4%); Veterans Health data showed a 0.86 hazard ratio for dementia; and UK Biobank data showed lower risk for all-cause (0.83 h), and vascular dementia (0.58 h) with a dose–response association by vaccination term. Randomized trials suggest fewer adverse cardiovascular events in vaccine recipients giving even more biological plausibility to this concept. Despite that, prevention through influenza vaccination is not fully realized in older adults due to low levels of perceived risk, vaccine confidence, and variations in clinical practice guidance. This public health perspective reviews the physiopathological and epidemiological evidence in support of influenza vaccination as a pragmatic, dementia risk–modifying intervention within healthy aging strategies and encourages the inclusion of vaccination status in hospital discharge and chronic-care pathways, integration of cognitive outcomes in monitoring, and equity-centered research to eliminate barriers to behavioral and implementation.

## Introduction: from respiratory prevention to cognitive preservation

The demographic reality of aging societies has heightened interest in strategies to delay or prevent dementia. While control of midlife risk factors is still primary, an adjunct, unused lever is in plain sight: seasonal influenza vaccination. This is a complementary, low-cost and scalable intervention which can be integrated into the existing healthcare pathways. Besides preventing a familiar, common respiratory infection, influenza vaccination may decrease vascular and neuroinflammatory assaults that promote cognitive decline. Growing population-based evidence links vaccination with decreased incident dementia, and randomized trials indicate that influenza vaccination decreased major adverse cardiovascular events—pathways strongly linked to late-life cognitive trajectories. This perspective combines physiopathological and epidemiologic reasoning to position influenza vaccination in the dementia-prevention pantheon, explores perception failures among older adults and healthcare workers, and outlines policy, organizational, and research strategies to convert evidence into outcomes.

## Influenza harms in older adults: more than a self-limited respiratory illness

Globally, influenza continues to be a significant source of excess morbidity and mortality, particularly among older adults. Modeling work estimates there are 291,000-646,000 seasonal influenza-associated respiratory deaths worldwide annually, with mortality rates increasing exponentially between ages 65–74 and age ≥ 75 years [1]. These significantly underestimate the overall burden, as influenza leads to non-respiratory complications that are likely to be particularly important for cognitive aging. In a landmark self-controlled study design with laboratory-confirmed cases, the risk of acute myocardial infarction was approximately six times greater in the first week after influenza infection, demonstrating a potent prothrombotic and proinflammatory initiator of vascular events [2]. Cerebral ischemia demonstrates similar vulnerability to infectious triggers, where at the population level vaccination (versus unvaccinated) against influenza is associated with lower stroke risk (pooled OR ~ 0.82) in meta-analysis [3]. Influenza-induced systemic inflammation could produce effects on the brain in the setting of no true neuroinvasion. In mice, non-neurotropic strains of influenza A virus induce prolonged microglial activation, synapse loss, and memory decline that persists long after viral clearance [4]. Overall, population and experimental evidence point towards a two-pronged pathway by which influenza infections may exacerbate cognitive decline in older adults: (i) by acting as initiators of acute cardio- and cerebrovascular events that contribute to pre-existing cerebral macro- and micro-vascular injury; and (ii) activating neuroinflammatory cascades that are gaining attention as potential mediators of neurodegeneration.

## Does influenza vaccination reduce dementia risk? The state of the evidence

Observational data have recently expanded in both scale and methodological rigor at a rapid pace. A recent 2023 meta-analysis of observational cohort studies (*n* ≈ 2.1 million, 4–13 years follow-up) found 31% less risk for dementia with influenza vaccination (pooled RR 0.69, 95% CI 0.57–0.83) [5]. Large-sample studies with careful matching confirm this association, even if residual confounding could affect the causality linkage due to the methodological design. In a claims-based cohort study of 935,887 propensity-score-matched pairs aged ≥ 65, prior influenza vaccination was recognized with a 40% relative risk reduction for incident Alzheimer’s disease over ~ 4 years (RR 0.60) and an absolute risk reduction of 3.4% (number needed to vaccinate ~ 29) [6]. However, as with other retrospective claims-based analyses, such designs may be susceptible to immortal time bias if vaccination status is not appropriately treated as a time-dependent exposure. More recent studies have explicitly addressed this limitation. In the Veterans Health Administration, time-to-event models with inverse-probability weighting on observational data indicated that vaccinated older adults had lower incidence of dementia (HR 0.86, 95% CI 0.83–0.88) with the strongest signal among participants who received multiple doses of vaccine across years and seasons [7]. Finally, a 2024 prospective UK Biobank analysis modeled vaccination as a time‑varying exposure, employed repeated covariate adjustment, and observed reduced risk of all‑cause dementia (HR 0.83, 95% CI 0.72–0.95) and vascular dementia (HR 0.58, 95% CI 0.39–0.86), with a dose–response across cumulative vaccinations [8]. Taken together, these later analyses mitigate concerns related to immortal time bias and strengthen confidence that the observed associations are not solely artefacts of study design. These findings address the limitations of immortal-time bias and residual confounding that can plague retrospective studies. Causation cannot be assured in observational datasets alone. Nevertheless, some features add plausibility: number of replications observed across health systems, evidence of dose-response [7]– [8], specificity of vascular dementia with prospective analysis [8], and a plausible mechanism via vascular and neuroinflammatory pathways. Accordingly, the strength of association from cohort studies (relative risk reductions of 14–40% across studies) is roughly similar to effect sizes for influenza vaccination on major vascular outcomes in randomized trials (discussed below), suggesting that the apparent cognitive benefit may be mediated largely by fewer vascular events and reduced inflammatory surges over time.

## Bridging mechanisms: vaccination, vascular events, and cognition

There has been strong evidence that cardiovascular benefits of the influenza vaccine are well-supported especially for these high-risk groups. A recent meta‑analysis of six randomized clinical trials (*n* = 9,001) found a 34% reduced risk of major adverse cardiovascular events (MACE) within one year of vaccination (RR 0.66), with stronger benefit among patients with recent acute coronary syndrome (RR 0.55). The number needed to vaccinate was ~ 56 to prevent one MACE overall; estimates suggest an NNT ~ 36 to prevent one cardiovascular death in higher‑risk patients [9]. The IAMI trial, a double-blind, multicenter RCT of patients post myocardial infarction, or at high-risk for coronary disease, had lower 12-month risks of the composite of all-cause mortality, MI, or stent thrombosis—and all cause and cardiovascular mortality—compared to placebo patients [10]. Observational studies and meta-analyses also suggest that influenza vaccination decreases risk of stroke [3], presumably through reduced infection induced thrombosis and endothelium activation.

With regard to dementia, the vascular effects are not peripheral. Vascular brain injury is the norm—even in clinically diagnosed Alzheimer’s disease—and the cumulative ischemic burden predicts steeper cognitive decline. Therefore, a seasonal intervention that mitigates myocardial infarction, heart failure exacerbations, and stroke would be expected to alter long-term cognitive course of trajectory. Moreover, consider too that vaccination likely works to prevent systemic inflammatory surges that travel to the brain, and the dementia signal seen in cohorts appears less a statistical oddity and much more like the sum of continuing and biologically meaningful protections cumulatively.

## Risk-perception gaps and barriers

If influenza vaccination likely decreases dementia, why do older adults access it poorly? Part of the answer relates to the ways people think and feel about vaccines. The WHO’s Behavioral and Social Drivers (BeSD) model describes that vaccination decisions are influenced by what people think and feel (confidence, risk appraisal), social dynamics (norms, trust), and practical matters (access, opportunity costs) [11]. A systematic synthesis of barriers specific to influenza vaccination identifies common themes: under-appraisals of influenza severity in older age; beliefs about low personal risk; hesitancy about efficacy or worry about side effects; and limited/non-tailored advice from health practitioners [12]. For healthcare personnel—trusted influencers whose uptake effects patient uptake—the same themes arise: worries about side effects, doubts about efficacy, low perceived risk, and implementation barriers [12]. The result is misalignment: older people and many clinicians (without awareness) consider the inconvenience of vaccination with short-term respiratory outcomes, and underestimate avoided vascular hospitalizations and potentially protected cognitive function. Coverage data from Europe illustrates the disparity from aspiration to reality. The European Union set a 75% coverage target for older adults; although some countries saw increases in 2020–21, most do not reach the target, and saw coverage rates decreasing in recent years [13]. The experience from the US and other locales is similar: even when reimbursement is adequate, access and advice practices differ and the opportunity for vaccination at hospital discharge or chronic disease management is missed.

## From association to action: policy and organizational levers

*Reconceptualize the value proposition*. Public messaging and clinical guides should honestly frame influenza vaccination as not just avoidance of winter’s respiratory illness but as a mechanism to reduce heart attacks, reduce strokes, protect the cognitive reserve and potentially delay cognitive decline. This reframing is in keeping with the evidence base and can be validated by meeting older adults in the place so that their priorities are on independence and cognition.

Dementia-prevention recommendations and primary-care cognitive health programs and projects should have seasonal influenza vaccination as a recommended, trackable intervention.

*Use the right vaccines for the right age groups*. Immunosenescence reduces vaccine effectiveness. Effectiveness can be improved by preferentially using higher-dose, adjuvanted, or recombinant vaccine formulations in people > 65. This is the recommendation of the U.S. Advisory Committee on Immunization Practices [14]. Health systems can align formularies, order procurement, and standing orders to make these formulations the default for older adults.

*Make vaccination the easy default across care pathways*. The strength and most consistently effective actions to improve vaccination uptake in older adults are practical: systematic reminders and recalls, standing orders, nurse- or pharmacist-initiated vaccination, and attempts to bring vaccination to the patient instead of the opposite. A Cochrane review of 61 trials in older adults concluded that client reminders (letters, postcards), personalized calls, and higher-intensity interventions (home visits by facilitator) improved vaccination uptake; team-based models, where nurses or pharmacists educate and vaccinate were very effective [15]. Influenza vaccination should be a standard discharge item for adults over 65 years of age being discharged from the hospital or post-acute care when admitted for cardiovascular, pulmonary, or cerebrovascular admissions; long-term care facilities would benefit from an opt-out standing order each year.

*Co-administer vaccination and opportunistic vaccination*. Opportunistic vaccination can decrease friction with routine visits, cardiac rehabilitation, pharmacy visits, and dialysis. If operational supports (e.g. scheduling, stock, documentation) would allow co-administration, it could condense multiple preventive interventions to one interaction — if operational supports (i.e. scheduling, stock and documentation) were able to be provided.

*Health communication linked to behavioral drivers*. Using the BeSD framework, interventions should not only emphasize reducing heart attacks, reducing strokes (confidence builders), but also include social norm cues (clinician and family uptake), and remove practical barriers (no appointment walk-ins, mobile clinics, home vaccination for homebound) for health clinics. For healthcare workers, the intervention should address the effectiveness and side effects with nimble, substantiated, succinct points based on evidence, and on-site convenient vaccination with visible leadership endorsement [12].

*Move beyond key coverage indicators*. Health systems and payers should allow inclusion of cognitive outcomes (e.g. incident dementia, trajectories for cognitive screening) and vascular hospitalizations on dashboard metrics on influenza vaccination. This would speed learning on real world benefits and help support the case for ongoing investment.

## Research directions

Pragmatic trials and emulations should include data related to the cognitive reserve. The vascular endpoint trials provide evidence of benefit [9]– [10]; integrating standardized cognitive screening and leveraging dementia diagnoses over years would measure downstream cognitive benefits. The UK Biobank analyses imply a dose-response across years [8]; trials and emulations should carefully examine if routine yearly vaccination provides cumulative neurocognitive benefit. Second, future studies should detail how vaccination alters neuroinflammatory set-points in older adults. To translate animal discoveries to humans, longitudinal profiles of peripheral cytokines, neurofilament light, and imaging indices of microglial activation over subsequent influenza seasons, with comparisons of vaccinated and unvaccinated cohorts, will be required. It is also testable whether vaccines developed with enhanced methods (high-dose, adjuvanted) yield larger or longer-lasting suppression of neuroinflammatory responses. Third, it is necessary to embrace an equity perspective. The largest burdens of dementia and influenza often occur in communities with inequalities and barriers to access. Future trials can juxtapose innovative care (e.g. pharmacy partnerships) versus usual care on rates of vaccination, events leading to vascular hospitalizations, and cognitive outcomes, with adaptive designs to vary intensity based on early response to intervention. Finally, health‑economic models should be updated to include vascular and cognitive objectives. Conventional influenza vaccine evaluations center on preventing respiratory illness; incorporating prevented cardiovascular events and delayed dementia onset would likely meaningfully shift value estimates toward targeted funding for older adults.

## Conclusion

Annual seasonal influenza vaccination is a safe, scalable intervention with benefits beyond the respiratory system. Influenza vaccination may reduce vascular events and dampen inflammatory spikes, which could contribute to slower cognitive decline; consistent associations have been observed across large cohorts, alongside randomized cardiovascular benefits. While these findings do not establish causality, they suggest that influenza vaccination may represent a biologically plausible and low-risk intervention with potential downstream cognitive benefits.

Future research should prioritize pragmatic randomized trials and well-designed quasi-experimental studies integrating cognitive outcomes alongside vascular endpoints, as randomized controlled trials with dementia-specific endpoints are currently lacking. Influenza vaccination should be framed as a complementary intervention within healthy cognitive aging strategies; use enhanced vaccines for adults ≥ 65 and embed opportunistic, low‑friction delivery into routine care. These steps can produce immediate population benefits as disease‑modifying dementia therapies evolve. The price of not acting is high, and the steps are reasonably simple. The migration of influenza vaccination from seasonal afterthought to centerpiece of healthy cognitive aging is a policy option available to pursue today.

This Perspective is narrative in nature and does not follow a systematic review methodology; accordingly, it does not claim exhaustive coverage of the literature but rather offers a reasoned interpretation of the most methodologically informative evidence, with explicit consideration of key limitations. Our policy recommendations are intended to be proportionate to the level of evidence, suggesting influenza vaccination as a low-risk, already recommended preventive intervention that may contribute to healthy cognitive aging when integrated within broader dementia-prevention frameworks.

## Data Availability

No datasets were generated or analysed during the current study.

## References

[1] Iuliano AD, Roguski KM, Chang HH, Muscatello DJ, Palekar R, Tempia S et al (2018) Global seasonal Influenza-associated mortality collaborator Network. Estimates of global seasonal influenza-associated respiratory mortality: a modelling study. Lancet 391(10127):1285–1300. 10.1016/S0140-6736(17)33293-2 29248255 10.1016/S0140-6736(17)33293-2 PMC5935243

[2] Kwong JC, Schwartz KL, Campitelli MA, Chung H, Crowcroft NS, Karnauchow T et al (2018) Acute myocardial infarction after Laboratory-Confirmed influenza infection. N Engl J Med 378(4):345–353. 10.1056/NEJMoa1702090 29365305 10.1056/NEJMoa1702090

[3] Lee KR, Bae JH, Hwang IC, Kim KK, Suh HS, Ko KD (2017) Effect of influenza vaccination on risk of stroke: A systematic review and Meta-Analysis. Neuroepidemiology 48(3–4):103–110. 10.1159/000478017 28628919 10.1159/000478017

[4] Hosseini S, Wilk E, Michaelsen-Preusse K, Gerhauser I, Baumgärtner W, Geffers R et al (2018) Long-Term neuroinflammation induced by influenza A virus infection and the impact on hippocampal neuron morphology and function. J Neurosci 38(12):3060–3080. 10.1523/JNEUROSCI.1740-17.2018 29487124 10.1523/JNEUROSCI.1740-17.2018 PMC6596076

[5] Sun H, Liu M, Liu J (2023) Association of influenza vaccination and dementia risk: A Meta-Analysis of cohort studies. J Alzheimers Dis 92(2):667–678. 10.3233/JAD-221036 36744343 10.3233/JAD-221036

[6] Bukhbinder AS, Ling Y, Hasan O, Jiang X, Kim Y, Phelps KN et al (2022) Risk of alzheimer’s disease following influenza vaccination: A Claims-Based cohort study using propensity score matching. J Alzheimers Dis 88(3):1061–1074. 10.3233/JAD-220361 35723106 10.3233/JAD-220361 PMC9484126

[7] Wiemken TL, Salas J, Hoft DF, Jacobs C, Morley JE, Scherrer JF (2021) Dementia risk following influenza vaccination in a large veteran cohort. Vaccine 39(39):5524–5531. 10.1016/j.vaccine.2021.08.046 34420785 10.1016/j.vaccine.2021.08.046

[8] Zhao Y, Meng X, He Q, Wang S, Wang X, Wang C et al (2024) Prospective cohort study evaluating the association between influenza vaccination and neurodegenerative diseases. Npj Vaccines 9:47. 10.1038/s41541-024-00841-z 38431710 10.1038/s41541-024-00841-z PMC10908860

[9] Behrouzi B, Bhatt DL, Cannon CP, Vardeny O, Lee DS, Solomon SD, Udell JA (2022) Association of influenza vaccination with cardiovascular risk: A Meta-analysis. JAMA Netw Open 5(4):e228873. 10.1001/jamanetworkopen.2022.8873 35486404 10.1001/jamanetworkopen.2022.8873 PMC9055450

[10] Fröbert O, Götberg M, Erlinge D, Christiansen EH, MacIntyre CR et al (2021) Influenza vaccination after myocardial infarction: A Randomized, Double-Blind, Placebo-Controlled, multicenter trial (IAMI). Circulation 144(18):1476–1484. 10.1161/CIRCULATIONAHA.121.057042 34459211 10.1161/CIRCULATIONAHA.121.057042

[11] World Health Organization (2022) Behavioural and social drivers of vaccination: tools and practical guidance for achieving high uptake. WHO, Geneva

[12] Schmid P, Rauber D, Betsch C, Lidolt G, Denker M-L (2017) Barriers of influenza vaccination intention and behavior—a systematic review of influenza vaccine hesitancy, 2005–2016. PLoS ONE 12(1):e0170550. 10.1371/journal.pone.0170550 28125629 10.1371/journal.pone.0170550 PMC5268454

[13] Del Riccio M, Guida A, Boudewijns B et al (2025) A missed opportunity? Exploring changes in influenza vaccination coverage during the COVID-19 pandemic: data from 12 countries worldwide. Influenza Other Respir Viruses 19(1):e70057. 10.1111/irv.70057 39778912 10.1111/irv.70057 PMC11710886

[14] Grohskopf LA, Alyanak E, Ferdinands JM, Broder KR, Blanton LH, Blanton L et al (2022) Prevention and control of seasonal influenza with vaccines: recommendations of the advisory committee on immunization Practices—United States, 2022–23 influenza season. MMWR Recomm Rep 71(1):1–28. 10.15585/mmwr.rr7101a1 36006864 10.15585/mmwr.rr7101a1 PMC9429824

[15] Thomas RE, Lorenzetti DL (2018) Interventions to increase influenza vaccination rates of those 60 years and older in the community. Cochrane Database Syst Rev 5:CD005188. 10.1002/14651858.CD005188.pub4 29845606 10.1002/14651858.CD005188.pub4 PMC6494593

